# Exploring the characteristics of gut microbiome changes in lung cancer patients and healthy controls

**DOI:** 10.1038/s41598-026-48560-w

**Published:** 2026-04-18

**Authors:** Peisheng Xu, Juan Chen, Xingbing Lu, Hongmei Luo

**Affiliations:** 1 Health Management Center, General Practice Medical Center, West China Hospital, Sichuan University, Chengdu, People’s Republic of China; 2 Department of Laboratory Medicine, West China Hospital, Sichuan University, Chengdu, People’s Republic of China; 3 Department of Ophthalmology, West China Hospital, Sichuan University, Guoxue Lane37, Chengdu, Sichuan People’s Republic of China; 4 West China School of Nursing, Sichuan University, Chengdu, People’s Republic of China

**Keywords:** Lung cancer, Gut microbiome, 16S rRNA sequencing, Biomakers, Biomarkers, Cancer, Microbiology, Oncology

## Abstract

Lung cancer is among the most prevalent and lethal malignant neoplasms worldwide. Although the role of the pulmonary microbiome in the pathogenesis of lung cancer has been examined, the structure, diversity, and composition of the gut microbiome in lung cancer remain largely unclear. The present study is chiefly concerned with the analysis of the characteristics and alterations of the gut microbiome in lung cancer patients and healthy individuals, and with the exploration of potential characteristic gut microbiome in lung cancer patients. Stool samples were collected from 40 patients with lung cancer and 20 healthy controls at the Lung Cancer Center of West China Hospital, Sichuan University. The samples were analysed using 16S rRNA gene amplicon sequencing to investigate differences in the relative and absolute abundance, diversity, and functionality of the gut microbiome between the two groups. The predominant gut microbiome communities in lung cancer patients and healthy controls were found to comprise Bacteroidetes, Firmicutes, Proteobacteria, and Actinobacteria. The F/B (Firmicutes/Bacteroidetes) ratio in the lung cancer group (0.73) was lower than that in the healthy control group (0.96). We found that there was no significant difference in α-diversity between LC patients and the control group (*p* > 0.05), whereas β-diversity of the gut microbiome revealed differences in microbial community structure between the patient and control groups (stress < 0.2). The healthy controls exhibited higher abundances of Bacteroidetes and Firmicutes as dominant bacterial groups, whereas the characteristic bacterial groups in lung cancer patients were the Ruminococcus_gnavus_group and the Prevotellaceae NK3B31 group. Studies have demonstrated that the gut microbiome of patients with lung cancer patients undergoes changes, and characteristic gut microbiome profiles may serve as potential diagnostic markers for lung cancer. Furthermore, we have revealed that impairments in normal gut microbiome function may be associated with the development and progression of lung cancer, providing valuable insights for the early prevention, diagnosis, and targeted intervention of lung cancer.

**Supplementary Information:**

The online version contains supplementary material available at 10.1038/s41598-026-48560-w.

## Introduction

Lung cancer (LC) is the most prevalent type of cancer worldwide, including within China, accounting for approximately 2.5 million new cases on a global scale, constituting 12.4% of all cancers. Furthermore, it is the leading cause of cancer deaths, with an estimated 1.8 million fatalities (18.7%)^1,2^. Currently, the overall 5-year survival rate for lung cancer patients is only 21.7%^3^, while the 5-year survival rate for patients with advanced disease is as low as 8%^4^. The pathogenesis of lung cancer and its risk factors remain largely unclear, primarily including exposure to secondhand smoke, radon exposure, air pollution, asbestos, and having a first-degree relative with lung cancer^4,5^. A plethora of research has indicated that LC is a multifaceted disease arising from the interaction between the host and various environmental factors. The relationship between dysbiosis and cancer has garnered extensive attention and exploration in the scientific community^6^. This underscores the pressing need for the expeditious diagnosis of lung cancer and the development of novel therapeutic approaches.

Recent technological developments in the field of cancer microbiome research, including 16S rRNA and metagenomics, have led to significant advancements in our understanding of the human gut microbiome. It is now recognised that this plays a crucial role in the initiation and progression of cancer, as well as in the response to chemotherapy and immunotherapy. This is due to the ability of the gut microbiome to modulate the tumour microenvironment and reshape the host immune response^7^. Wang et al.^8^ identified characteristic bacteria, including Streptococcus, *Haemophilus influenzae*, and *Corynebacterium minutae*, in bronchoalveolar lavage fluid (BALF) from 75 lung cancer patients. This study established a diagnostic model capable of distinguishing lung cancer from benign lesions. Studies have documented a significant increase in microorganisms such as Veillonella and Streptococcus in the saliva/sputum of lung cancer patients^9^. The gut microbiome has been demonstrated to modulate immunity across entire organ systems. In recent years, the crucial role of gut microbiome–host interactions in the development and treatment of lung cancer has attracted increasing attention. The gut microbiome also plays a pivotal role in several hallmark features of cancer, including angiogenesis, invasion, the tumour immune microenvironment, and apoptosis^10^. Researchers have demonstrated that by regulating the tumour microenvironment through human-to-mouse gut microbiome transplantation experiments, it is possible to influence tumour growth and immune infiltration^11^. Zheng et al.^12^ analyzed the gut microbiome of early-stage LC patients, revealing the microbial profiles characteristic of LC patients and establishing predictive models. The human gut microbiome has been shown to modulate the efficacy of immunotherapy and anti-cancer immune surveillance by forming pathogen-associated molecular patterns (PAMPs), antigens, and metabolites that activate or suppress immune cell populations^13^. Researchers have established mouse models to investigate the relationship between microbiota-immune interference and the development of liver cirrhosis^14^. It has been hypothesised that the gut microbiome may influence LC proliferation and the immune microenvironment by modulating immune inflammatory responses. The underlying mechanisms remain unclear and require further investigation.

Despite the proposal of the “gut–microbiome–lung” axis hypothesis by scholars in the field and the subsequent conduct of related research, the precise mechanisms underpinning this phenomenon remain to be elucidated. The evidence base regarding the precise characteristics of the microbiota and metabolic pathways linking the gut microbiome to LC is limited, and further investigation is required to determine potential influencing mechanisms. It is imperative to comprehend the composition of the gut microbiome in LC patients and its impact on these individuals. Therefore, a cross-sectional study was conducted in which fecal samples were collected from individuals diagnosed with LC and from healthy controls. The samples were then analysed in order to conduct a comparative study of the gut microbiome. The aim was to explore any differences in gut microbiome composition that could be associated with LC, and to consider the implications of these differences.

## Materials and methods

### Study participants

A total of 60 faecal samples were collected from 40 LC patients (median age: 61.9 years) and 20 age- and sex-matched healthy controls from the Lung Cancer Centre, West China Hospital, Sichuan University. The diagnosis of all LC patients was made according to their histopathological features, with the use of the Tumor Node Metastasis (TNM) scale for the classification of malignant tumours following surgical intervention. Prior to the collection of samples, none of the patients had undergone chemotherapy, radiation therapy, targeted therapy, immunotherapy, or surgery for lung cancer. Lung cancer patients and healthy individuals with one or more of the following conditions were excluded from the study: congestive heart failure, irregular sleep patterns, long-term smoking, alcohol abuse, respiratory failure, intestinal diseases, renal failure, severe liver dysfunction, and use of probiotics or antibiotics within one month prior to sample collection. For patients diagnosed with lung cancer, following their initial outpatient visit, a series of dietary and lifestyle modifications were implemented. These included the cessation of smoking and alcohol consumption, the discontinuation of antibiotic use, and the adherence to a healthy, regular sleep schedule for a period of at least one month. The rationale behind these modifications was to minimise the impact of potential confounding factors on the gut microbiota. Furthermore, for healthy individuals undergoing routine health checkups, the following screening criteria were established: a light diet, a healthy and regular sleep schedule, non-smoking, non-alcohol consumption, and non-use of antibiotics or dietary supplements. The clinical characteristics of all participants are shown in Table 1. No statistically significant differences were observed in terms of age, BMI, and gender distribution between the groups (*p* ≥ 0.05). Prior to participation in the study, each patient provided written informed consent. The protocol was approved by the ethics committee of the West China Hospital, Sichuan University.

**Table 1:** Baseline characteristics of the discovery cohort.

	Patients with lung cancer (n = 40)	Haelth Control (n = 20)	*p* value*
Demographics/anthropometric			
Age year (mean ± SD)	61.92 ± 9.38	57.6 ± 10.14	0.119
Male/female (No.)	21/19	12/8	0.349
BMI (kg/m^2^) (mean ± SD)	23.04 ± 2.30	22.97 ± 3.31	0.929
Tumor type (%)			
ADC	20 (50.00%)	N/A	
SCC	20 (50.00%)	N/A	
TNM (%)			
T1	11 (27.50%)	N/A	
T2	11 (27.50%)	N/A	
T3	12 (30.00%)	N/A	
T4	6 (15.00%)	N/A	
TNM (%)			
N0 & N1	26 (65.00%)	N/A	
N2 & N3	14 (35.00%)	N/A	
Tumor metastasis (%)			
Metastasis	7 (17.5%)	N/A	
Nonmetastasis	33 (82.50%)	N/A	

*TNM* tumor node metastasis scale. Unpaired t-test was used to compare age and BMI between lung cancer group and healthy controls; Fisher’s exact test was used to compare gender distribution between the two groups. N/A, not applicable.

### DNA extraction and PCR amplification

Approximately 1 g of fresh faecal samples was collected into sterile EP tubes from all participants. Subsequent to collection, the fecal samples were frozen in liquid nitrogen and stored in a − 80 °C freezer until DNA extraction. The isolation of bacterial DNA from the fecal samples was conducted using a DNeasy PowerSoil kit (Qiagen, Hilden, Germany), with the procedure undertaken in strict accordance with the manufacturer’s guidelines. The concentration and purity of the DNA were measured using a NanoDrop 2000 spectrophotometer (Waters Scientific, Worms, Massachusetts, USA) and agarose gel electrophoresis, respectively. The extracted DNA was stored at a temperature of − 20 °C. The bacterial 16S rRNA gene was amplified using PCR, with the genomic DNA extracted earlier serving as the template. The amplification was performed using primers that were specific to the bacterial 16S rRNA gene and which were labelled with a barcode, in addition to Takara Ex Taq high-fidelity polymerase. The total amount of DNA fragments produced was approximately 1 μg, which was then used for library preparation. The amplification of the V3–V4 hypervariable regions of the bacterial 16S rRNA gene was conducted within a 25 μl reaction environment, utilising universal primer pairs (343F: 5′-TACGGRAGGCAGCAG-3′; 798R: 5′-AGGGTATCTAATCCT-3′)^15^. The reverse primer contained a sample barcode, and both primers were connected with an Illumina sequencing adapter.

### Library construction and sequencing

The quality of the amplicons was then subjected to visualisation through the process of gel electrophoresis. The PCR products were then purified using Agencourt AMPure XP beads (Beckman Coulter Co., USA) and quantified using a Qubit dsDNA assay kit. Subsequently, the concentrations were recalibrated to align with the requirements of the sequencing process. The sequencing process was conducted on an Illumina NovaSeq 6000 instrument, employing two paired-end read cycles of 250 bases each (Illumina Inc., San Diego, CA; OE Biotech Company, Shanghai, China).

### Pathway analysis

The abbreviation COG is an acronym for Clusters of Orthologous Groups of proteins. The proteins that comprise each COG are hypothesised to have descended from a single ancestral protein, and are thus either orthologs or paralogs^16^. Orthologs are defined as proteins from different species that have evolved through vertical descent (speciation) and typically retain the same function as the ancestral protein. Paralogs are defined as proteins within a given species that originate from gene duplication and may evolve new functions related to the original. A review of the homepage and accompanying documentation reveals that COG is an NCBI database^16^. The Chinese interpretation of COG is “clusters of orthologous proteins”. COG is categorised into two distinct classifications: one for prokaryotes and the other for eukaryotes. The prokaryotic category is generally referred to as the COG database. Statistical tests are to be performed on the predicted COG results (using the Wilcoxon test for two groups and the Kruskal–Wallis test for multiple groups), and the top 30 significant results are to be selected to generate a COG bar plot.

### Statistical analysis

All statistical calculations were performed in R3.4.3. The Z-score in the heat map is derived by first subtracting the mean of the control group and then dividing the standard deviation of the control group. The Z-score is negative when the raw value is below the mean and positive when above the mean. Fisher’s exact test was performed on categorical variables. The chi-square test was employed for the analysis of categorical variables. The Student’s t-test or Mann–Whitney Wilcoxon test is utilised to ascertain the significant difference between two groups. The fold-change was calculated by the ratio of means between pairwise comparisons. A *p* value of ≤ 0.05 indicates a statistically significant difference.

### Bioinformatic analysis

Paired-end reads were subjected to preprocessing using Trimmomatic software^17^, with the aim of detecting and removing ambiguous bases (N). Furthermore, the utilisation of a sliding window trimming approach enabled the elimination of low-quality sequences, characterised by an average quality score below 20. Following the trimming process, paired-end reads were assembled utilising FLASH software. The assembly was characterised by the following parameters: The parameters for the analysis were set at 10 base pairs of minimal overlapping, 200 base pairs of maximum overlapping, and 20% of maximum mismatch rate. Further denoising of the sequences was performed in the following manner: reads that were characterised by ambiguous, homologous sequences or a length of less than 200 bp were abandoned. The analysis revealed that 75% of bases with a frequency above Q20 were successfully retained using QIIME software^19^. Subsequently, reads containing chimeric sequences were identified and eliminated through the utilisation of VSEARCH^20^. Clean reads were subjected to primer sequences removal and clustering to generate operational taxonomic units (OTUs). All representative reads were subjected to annotation and subsequently blasted against the Silva database (Version 123) using the RDP classifier (with a confidence threshold of 70%). The 16S rRNA gene amplicon sequencing and analysis were conducted by OE Biotech Co., Ltd. (Shanghai, China).

## Results

### The ratio of gut microbiome composition between LC and control group

**Fig. 1:**
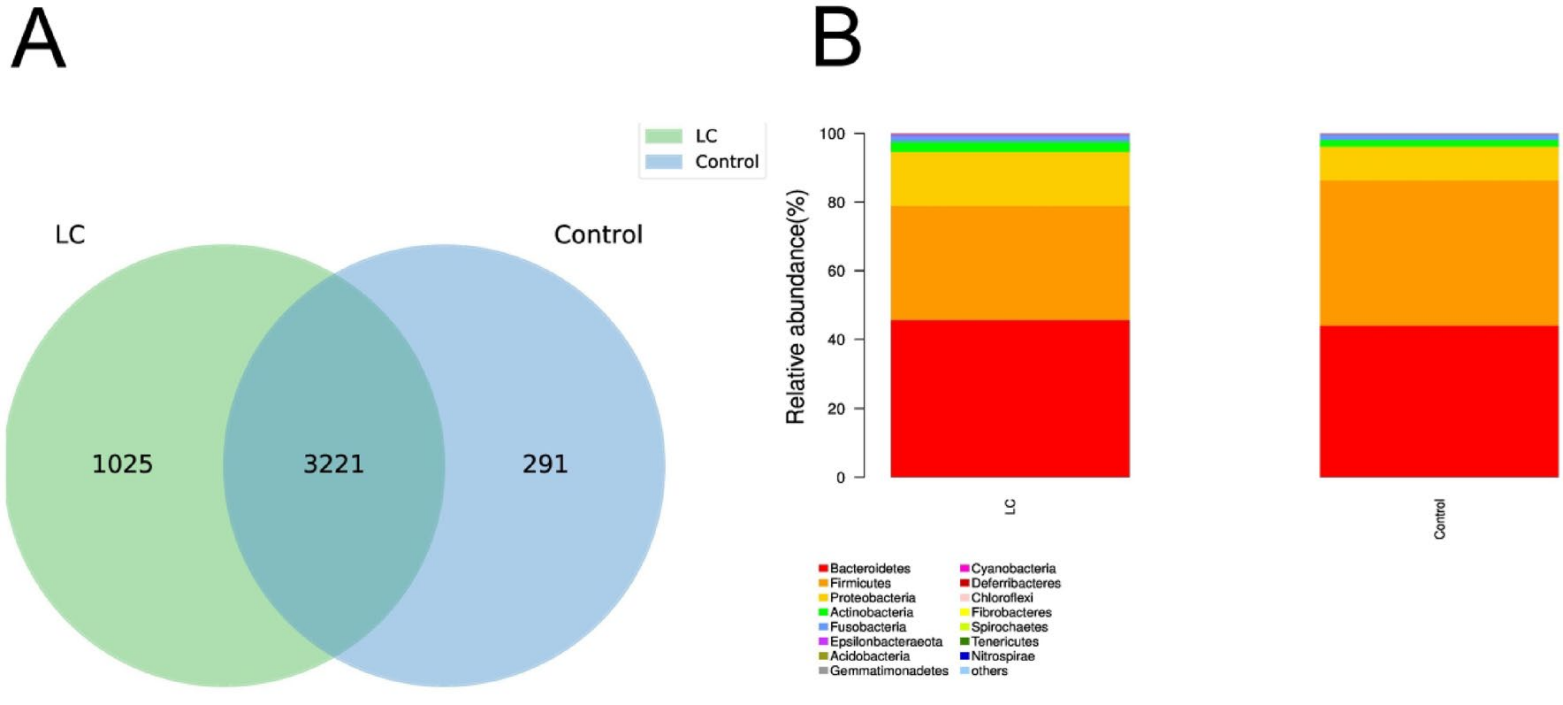
The ratio of gut microbiome composition between LC and Control group. (**A**) A Venn diagram visually illustrates the OTUs shared and unique to different sample groups. Each circle represents a group. The number inside each circle indicates the total number of OTUs in that group. The overlapping area represents the OTUs shared by both groups. The non-overlapping area represents the OTUs unique to that group. (**B**) Bar charts are used to illustrate the composition of different gut microbiota communities. Each bar represents a sample, and different colors indicate different annotation information. “Others” refers to all species other than those in the “Top” category.

The Venn diagram illustrates that a total of 4537 OTUs were identified following the combination of the HC and LC groups for analysis. Of these, 3221 OTUs were shared by both groups, 291 OTUs were present only in the HC group, and 1025 OTUs were present only in the LC group. The increase in the number of OTUs specific to the LC group suggests that new microbial taxa may have been introduced into the LC group (Fig. 1A). The gut microbiome of the LC and HC groups were primarily composed of the phyla Bacteroidetes, Firmicutes, Proteobacteria, and Actinobacteria, which accounted for over 85% of the total sequences (Fig. 1B, Table S1). The abundance of Bacteroidetes was found to be higher in the LC group than in the HC group, while the abundance of Firmicutes was found to be higher in the HC group than in the LC group. The F/B (Firmicutes/Bacteroidetes) ratios for the LC and HC groups were 0.73 (0.3319/0.4561) and 0.96 (0.4221/0.4401), respectively (Fig. 1B, Table S1).

### The alpha diversity of the gut microbiome

**Fig. 2:**
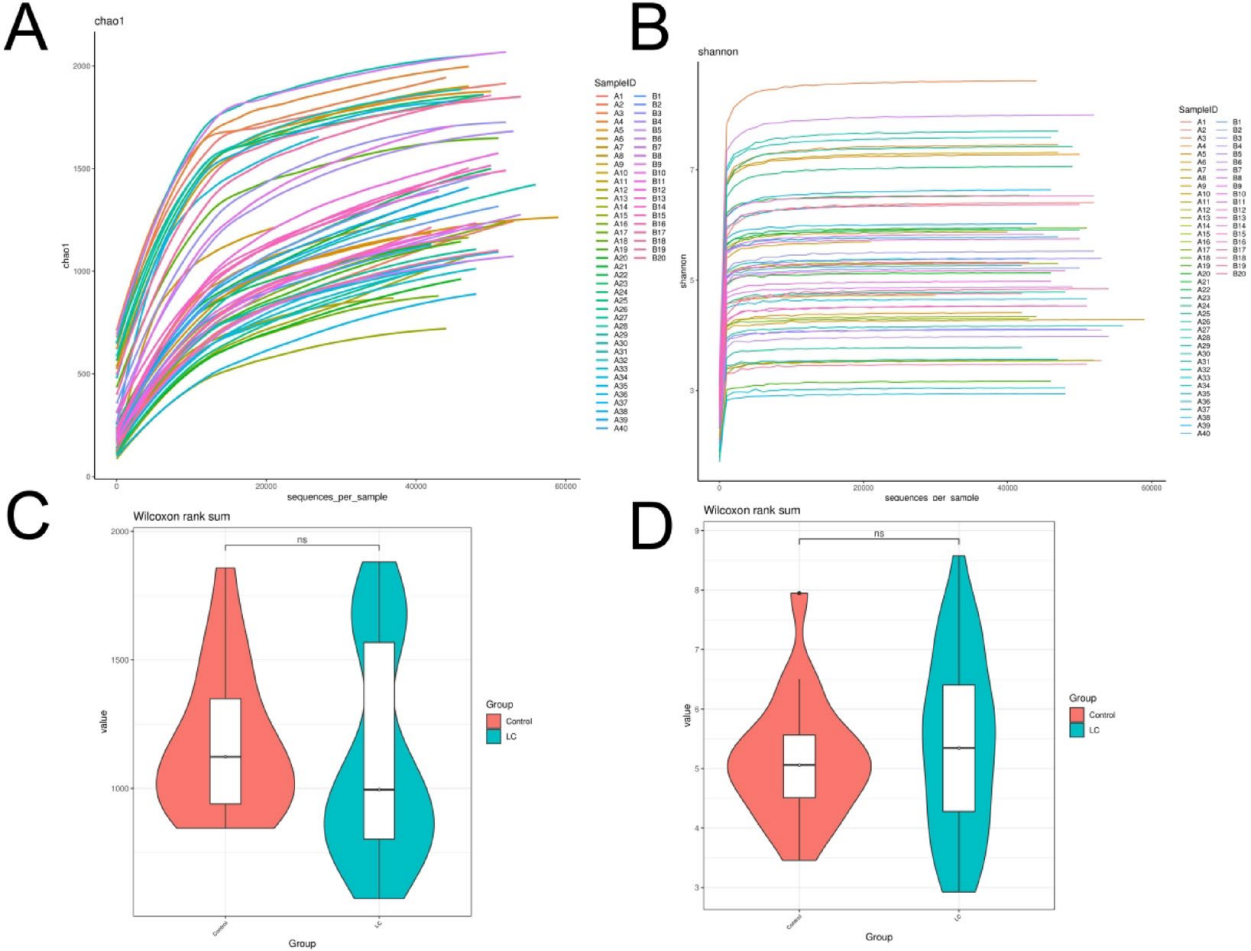
The comparison of gut microbiome alpha diversity between each group. *ns* no significance, *LC* lung cancer. (**A**,**B**) As the depth of sequencing increases, the diversity present in the dilution curve gradually increases; however, once the sequencing depth reaches a certain level, the curve experiences a flattening effect, which is indicative of the attainment of saturation in the data. (**C**,**D**) The boxplot of the α diversity index is a visual representation of the distribution of the diversity index across groups and the identification of any significant differences in the diversity index between groups. The x-axis denotes group classification, with green signifying the LC group and orange denoting the HC group; the y-axis represents the index values. ns: no statistically significant difference was observed.

The Chao1 and Shannon diversity indices demonstrate a gradual increase in diversity with increasing sequencing depth. When the sequencing depth reaches a certain level, the curve exhibits a flattening trend, which is indicative of data saturation (Fig. 2A,B). To evaluate alteration in the gut microbiome community structure between each group, the microbial alpha diversity was measured. The alpha diversity of chao1 (*p* > 0.05), and Shannon (*p* > 0.05) were no significant difference between LC patients and healthy controls (Fig. 2C,D).

### The β diversity analysis of the gut microbiome

**Fig. 3:**
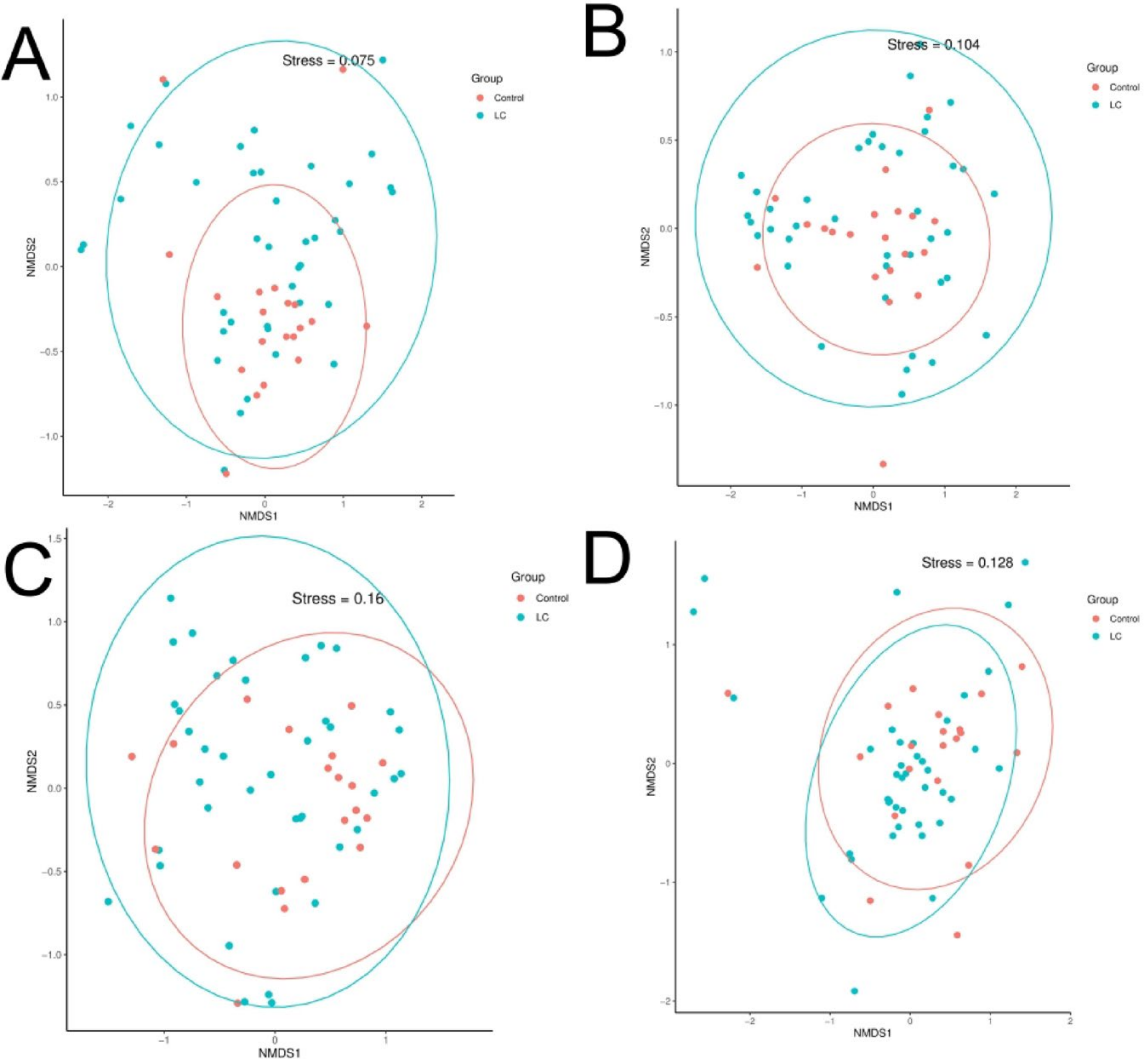
Graphics of different colors and shapes represent different samples. The closer the sample point are, the more similar the composition of the sample species is. Horizontal and vertical coordinates represent relative distance and have no practical significance. It is generally believed that the stress < 0.2 can be expressed by the two-dimensional point model of NMDS, and its figure has a certain explanatory significance. LC, lung cancer. (**A**–**D**) These principal coordinate analysis plots, based on weighted_unifrac, unweighted_unifrac, Bray–Curtis, and Euclidean distances, show whether there is a significant separation in microbial community structure between the LC group (blue) and the control group (red).

In order to evaluate the microbial community composition and the significance of distribution differences among the constituent communities, the microbiome of β diversity was measured. As a dimensionality reduction-based approach, NMDS (Nonmetric multidimensional scaling) analysis was applied for dissimilarities in the microbial composition between LC patients and healthy controls. The principal coordinate analysis plot has been derived from weighted_unifrac distances. A partial separation in the microbial community structures was observed between the LC group (blue) and the HC group (red). The PERMANOVA analysis demonstrated that the group factor accounted for 2.6% of the observed community variation. However, this result did not attain statistical significance (R^2^ = 0.02604, *p* = 0.155, stress = 0.075; Fig. 3A). The principal coordinate analysis plot has been created using the unweighted_unifrac distance metric. A significant divergence in microbial community structures was observed between the LC and HC groups. The PERMANOVA analysis demonstrated that the group factor accounted for 2.9% of the observed community variation, a result that was deemed to be statistically significant (R^2^ = 0.02902, *p* = 0.033, stress = 0.104; Fig. 3B). The principal coordinate analysis plot has been developed based on the Bray–Curtis distance. A partial separation in the microbial community structures between the LC and HC groups was observed. The PERMANOVA analysis revealed that the group factor accounted for 2.7% of the observed community variation. However, this result did not attain statistical significance (R^2^ = 0.02727, *p* = 0.063, stress = 0.16; Fig. 3C). The principal coordinate analysis plot has been developed on the basis of the Euclidean distance. A partial separation in the microbial community structures between the LC and HC groups was observed. The PERMANOVA analysis demonstrated that the grouping factor accounted for 2.0% of the community variation and was statistically significant (R^2^ = 0.02078, *p* = 0.225, stress = 0.128; Fig. 3D).

### Comparison of gut microbiome between the LC and HC groups

**Fig. 4:**
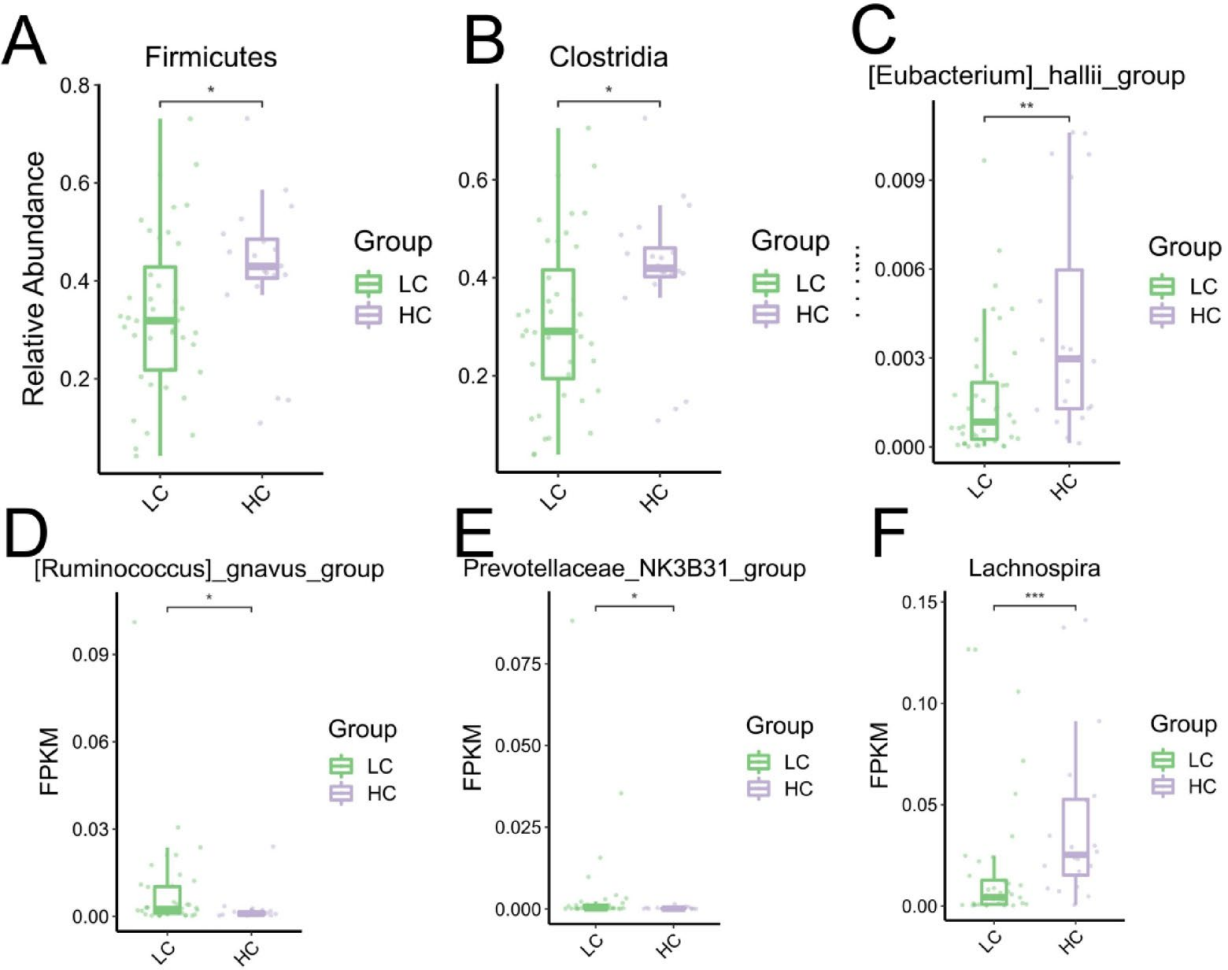
Difference in the abundance of gut microbiome between lung cancer and healthy controls. The distributions of taxa at phylum, class, and genus levels were based on the number of readings post-filtering and rarefying. The abundance of each group was plotted as log10 scale on the y axis. (**A**) Comparison of phylum level. (**B**) Comparison of classlevel. (**C**–**F**) Comparison of genus levels. *p* values were calculated using Two-tailed Kruskal Wallis test.*p < 0.05,**p < 0.01,***p < 0.001.

A substantial increase in abundance was detected for the Firmicutes, and Clostridia in the control group (Fig. 4A,B, *p* < 0.05). At the genus level, Lachnospira (Fig. 4F, *p* < 0.001), and *Eubacterium hallii* group (Fig. 4C, *p* < 0.01) were increased in the control group, while Prevotellaceae NK3B31 group (Fig. 4E, *p* < 0.05) and *Ruminococcus gnavus* group were significantly elevated in the LC group (Fig. 4D, *p* < 0.05).

### Specific species in multi-level tests

**Fig. 5:**
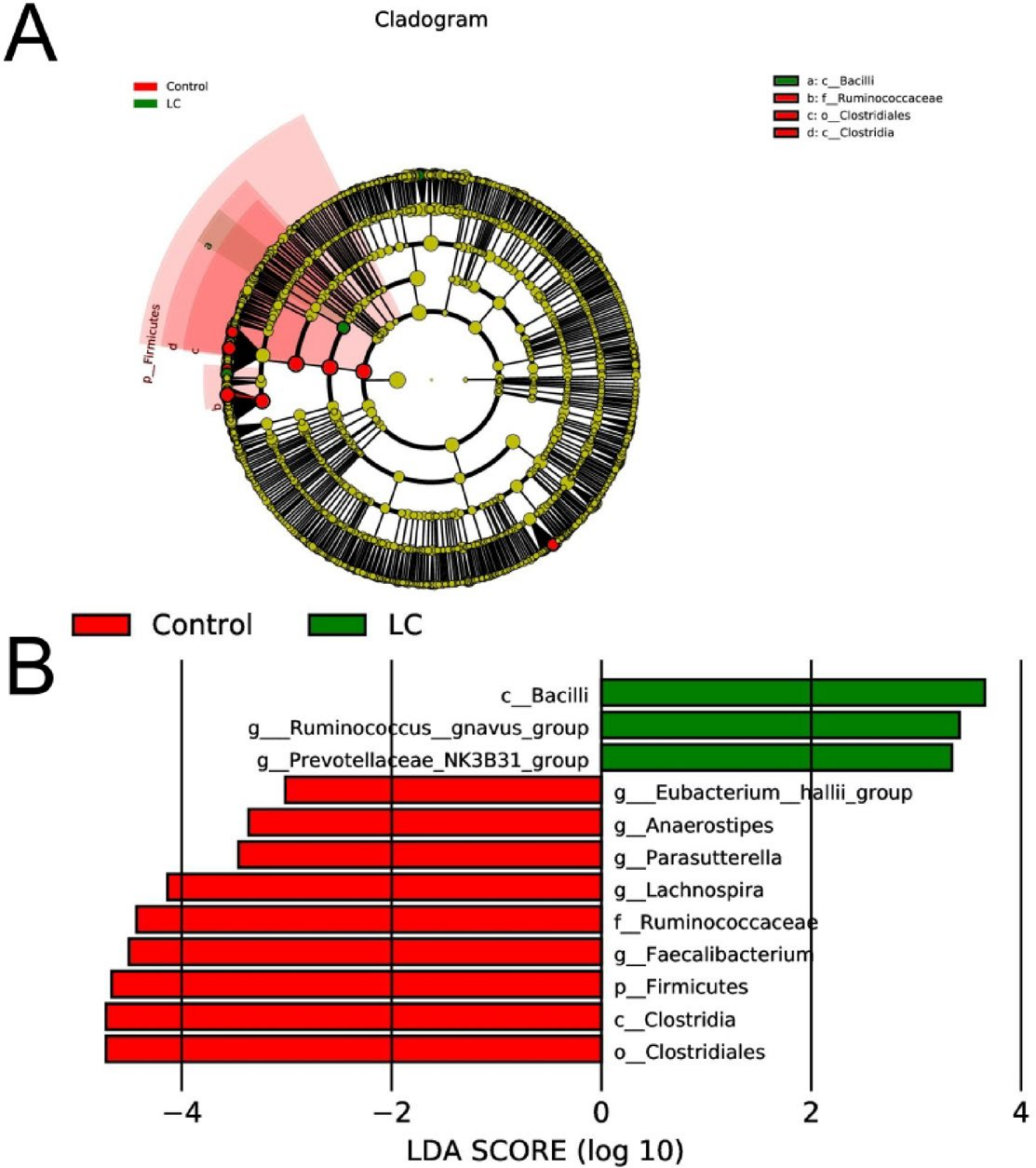
LEfSe discriminant analysis of species differences. (**A**) The significantly different species are shown in the cladogram. Each circle represents phylogenetic level from phylum to genus inside to outside. The diameter of each circle is proportional to the abundance of the taxon, and the colour of the circle indicates the biomarker. The length of the column represents the influence of significantly different species in relative abundance. (**B**) The LDA score obtained by linear regression analysis (LDA), the larger the LDA score, the greater the influence of species abundance on the difference effect. *LC* lung cancer.

The multi-level LEfSe (linear discriminant analysis (LDA) coupled with effect size measurements, LEfSe) analysis for biomarkers was used to distinguished the healthy group from the LC group, and to seek the potential bacterial biomarkers of the LC group. Multilevel LEfSe biomarker analysis revealed significant microbial dysbiosis between LC patients and healthy controls, indicating marked differences between the two groups. The elevated levels of Firmicutes, Clostridiales, Clostridia, Ruminococcaceae, Faecalibacterium, Lachnospira, Anaerostipes, Parasutterella, and Eubacterium__hallii_group were detected as markers in healthy controls (Fig. 5, *p* < 0.05). Increased gut microbiome such as Bacilli, Prevotellaceae_NK3B31_group, and Ruminococcus_gnavus_group genus were detected as markers in the LC group (Fig. 5, *p* < 0.05).

### Differential microbiome functional abundance spectrum in the LC group

We conducted a 16S functional prediction analysis utilising COG (Cluster of Orthologous Genes) functional annotation, with the descriptive information and functional data of each COG extracted from the egg NOG database. Compared to the healthy controls, results showed a significant decline in the functional abundance spectrum including 24 gut microbiota metabolic pathways in LC patients (Table 2). Of these, the expression of functional proteins involved in defence mechanisms decreased by more than 9%. Concurrently, the functional abundance profile of LC patients exhibited a substantial increase in seven pathways, with the expression of functional proteins implicated in extracellular structures and RNA processing and modification both rising by more than 32% (Table 2).

**Table 2:** Functional abundance spectrum of gut microbiome in LC patients and healthy controls.

Functional level classification	LC	Control	Ratio (%)
Defense mechanisms	1,010,609.05	1,120,193.40	− 9.78
Replication, recombination and repair	2,412,598.12	2,596,893.50	− 7.09
Signal transduction mechanisms	2,003,972.72	2,140,787.05	− 6.39
Cell cycle control, cell division, chromosome partitioning	466,190.55	496,517.05	− 6.10
Cytoskeleton	3191.70	3347.65	− 4.65
Translation, ribosomal structure and biogenesis	2,225,496.50	2,333,358.10	− 4.62
Transcription	3,198,621.25	3,345,509.35	− 4.39
Nucleotide transport and metabolism	1,049,688.47	1,093,495.20	− 4.00
General function prediction only	4,252,143.67	4,407,241.70	− 3.51
Cell wall/membrane/envelope biogenesis	2,592,900.42	2,686,559.55	− 3.48
Carbohydrate transport and metabolism	3,564,678.62	3,635,330.60	− 1.94
Coenzyme transport and metabolism	1,603,598.92	1,634,436.30	− 1.88
Lipid transport and metabolism	946,813.47	963,420.00	− 1.72
Amino acid transport and metabolism	3,036,371.45	3,063,928.40	− 0.89
Posttranslational modification, protein turnover, chaperones	1,163,270.87	1,166,017.80	− 0.23
Energy production and conversion	2,032,970.77	2,037,232.20	− 0.20
Chromatin structure and dynamics	2763.82	2730.85	1.20
Inorganic ion transport and metabolism	1,932,012.72	1,898,973.70	1.739
Intracellular trafficking, secretion, and vesicular transport	838,119.87	799,424.75	4.84
Secondary metabolites biosynthesis, transport and catabolism	476,321.47	438,771.10	8.55
Cell motility	471,714.42	421,722.85	11.85
Extracellular structures	1014.97	764.40	32.784
RNA processing and modification	3409.60	2551.55	33.62

Ratio (%) = (LC patients (median) − Healthy Controls (median))/Healthy Controls (median); *LC* lung cancer.

## Discussion

**Fig. 6:**
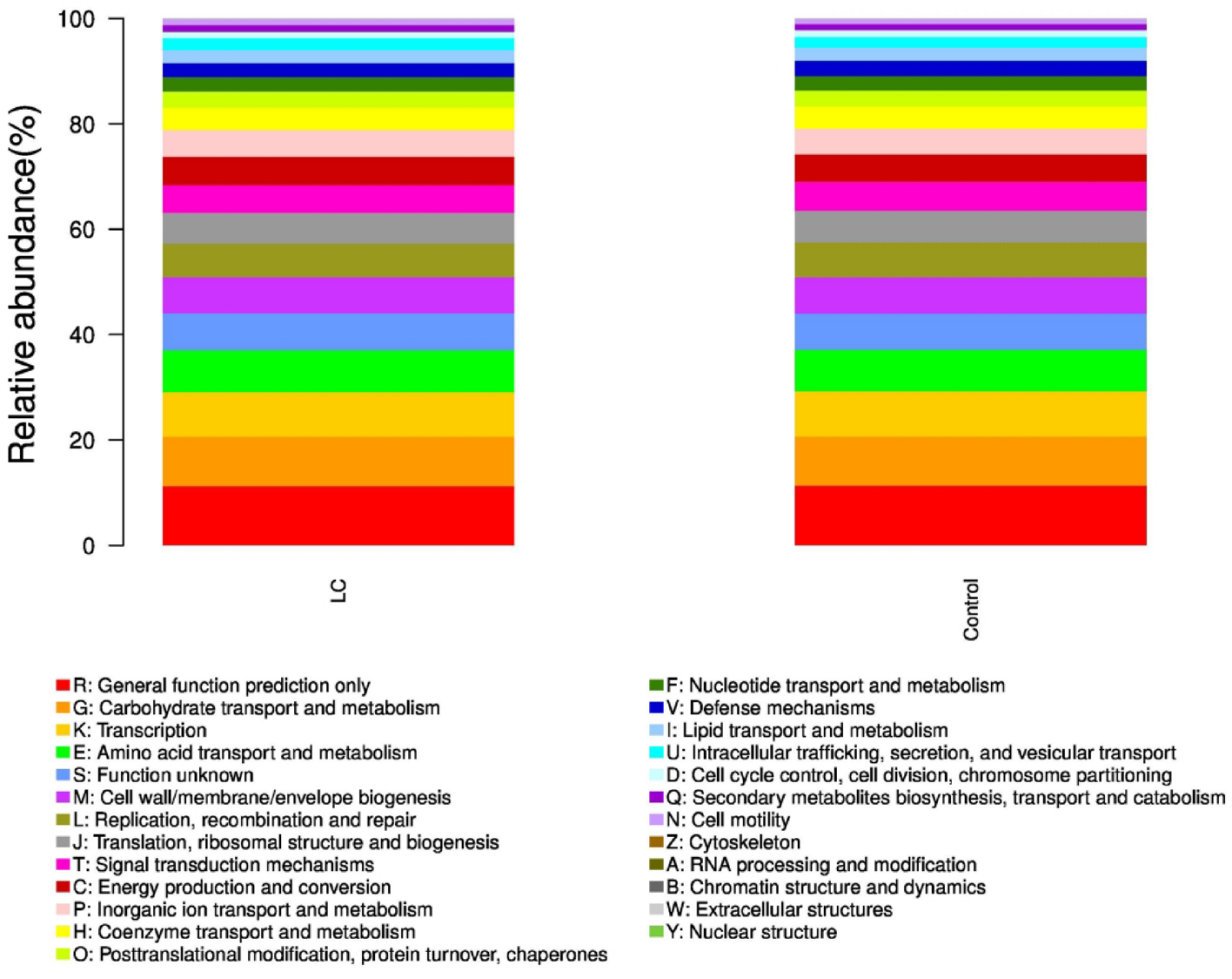
Functional abundance spectrum of gut microbiome in LC patients and healthy controls.

It was determined that both the healthy control group and the LC patient group were primarily composed of Bacteroidetes, Firmicutes, Proteobacteria, and Actinobacteria, with no significant differences observed in community composition or abundance (Fig. 1). The four aforementioned phyla represent dominant communities in both groups, playing crucial roles in maintaining gut diversity and human health. The study hypothesises that the Firmicutes/Bacteroidetes ratio (F/B) in the gut microbiome may serve as a potential predictive indicator for monitoring disease activity and treatment response in inflammatory bowel disease^21^. In our study, the gut microbiome of patients with LC demonstrated a significantly increased abundance of Bacteroidetes, accompanied by a lower F/B ratio in comparison to the healthy group (0.72 vs. 0.96) (Table S1). It is hypothesised that the F/B ratio may play a crucial role by influencing the gut microbiome composition and inflammatory response in LC, potentially serving as a biomarker for disease progression and a therapeutic target. The present study has demonstrated that breast cancer is characterised by elevated levels of Firmicutes and reduced levels of Bacteroidetes at the portal level. This finding constitutes a risk factor for breast cancer^22^. A mounting evidence base indicates that Actinobacteria, specifically probiotics such as Bifidobacteria, may have a role in the prevention of colorectal cancer (CRC). Furthermore, these bacteria could serve as a means to reduce pro-carcinogenic inflammatory markers in high-risk CRC patients^23^. The ongoing discovery of Actinobacteria may lead to the development of novel therapeutic strategies, particularly probiotic-based interventions aimed at preventing or managing gastrointestinal cancers^24^. This finding underscores the promise of probiotics as a preventative measure for high-risk populations, with the potential to augment the effectiveness of conventional cancer treatments by modulating gut microbiome composition. Furthermore, Proteobacteria have been demonstrated to play multifaceted roles in gastrointestinal cancers, with specific species stimulating inflammation, producing genotoxic metabolites, and impairing immune function. While there is evidence to suggest that pathogenic Proteobacteria increase cancer risk, recent studies suggest that targeted use of benign strains may offer therapeutic potential for maintaining gut health and preventing cancer^24^ (Fig. 6).

In our study, we found that patients with LC had no difference in gut microbial alpha diversity but showed significant differences in microbial composition compared to healthy controls (Figs. 2 and 3). This finding suggests that the composition and abundance of the gut microbiota in patients with LC are comparable to those in healthy individuals. α diversity has been demonstrated to be a reliable indicator of changes in gut microbiome community structure and intra-sample diversity. A substantial body of research has indicated that a highly diverse gut microbiome is conducive to human health. Conversely, a reduced gut microbiome abundance has been demonstrated to correlate with disease states^25^. Reduced gut microbiome diversity has been demonstrated to compromise immune function and systemic homeostasis, thereby influencing cancer progression and treatment outcomes. Furthermore, an enhancement in the response to anti-PD-1 therapy was observed in patients with NSCLC, which was significantly associated with increased gut microbiome α diversity^26^. In this study, the differences in gut microbiome α diversity between lung cancer patients and the healthy control group were not statistically significant. The analysis of β diversity was used to evaluate the composition of the gut microbiome and the significance of distribution differences among constituent communities. The NMDS exhibited distinct clustering between the LC and healthy groups. The results of this study suggest that healthy individuals may possess richer gut microbial diversity and more intact microbial structures, which are more conducive to intestinal health. The diversity of the gut microbiome has been demonstrated to correlate positively with enhanced immune responses and improved immunotherapy efficacy in patients with NSCLC^27^. Those demonstrating clinical benefit (defined as no disease progression for a minimum of 6 months) have been shown to exhibit higher gut microbiome diversity^28^. The gut microbiome associated with immunotherapy benefits may enhance antitumor immunity by modulating favourable immune responses. Conversely, an unfavourable composition of the gut microbiome may promote immunosuppressive mechanisms or microbial functions that support tumours^29^.

LEfSe analysis in this study revealed that Firmicutes exhibited lower abundance in the gut microbiome of all LC patients but was significantly elevated in healthy individuals, consistent with findings reported by Liu et al.^30^. Firmicutes represents one of the most prevalent bacterial phyla within the normal human gut, compassing all butyrate-producing bacteria. The metabolites of these bacteria, such as butyrate and other short-chain fatty acids (SCFAs), have been shown to exert a direct effect on intestinal epithelial cells, thereby contributing to the maintenance of gut integrity. In addition, these SCFAs have been observed to regulate the immune system by modulating G protein-coupled receptors and histone deacetylases. Furthermore, the anti-inflammatory and anti-tumour properties of these metabolites have been demonstrated, suggesting a potential for therapeutic applications in related diseases^31^. Firmicutes establish a mutualistic symbiosis with the host through innate tolerance and resistance, capable of regulating systemic immunity without inducing immunopathology. Intestinal processing of Firmicutes glycoconjugates has been shown to stimulate macrophages by releasing the cytokine IL-34 through diffusion, thereby enhancing defences against pneumonia, sepsis and meningitis^32^. Although butyrate possesses anti-inflammatory effects, under certain inflammatory conditions, high levels of butyrate-producing Firmicutes bacteria promote tumor growth by activating immune checkpoints that suppress antitumor immunity^33^. Firmicutes have been observed to exhibit dual roles in influencing immune responses, either suppressing or promoting cancer progression, depending on the balance of the surrounding gut microbiome. Faecalibacterium and Lachnospira are members of the SCFA-producing bacterial community, which has been demonstrated to exert positive effects on host immune function, intestinal barrier integrity, and mucus production. It has been demonstrated that these cells can induce regulatory T cells (Tregs) to exert anti-inflammatory and anti-tumour functions^34^. Moreover, their abundance is significantly reduced in breast cancer patients^22^. In the present study, the abundance of Faecalibacterium and Lachnospira in LC patients was found to be significantly reduced. This finding suggests that these bacterial communities may play a crucial role in protecting human immune function and reducing or delaying the occurrence and progression of tumour diseases and inflammatory responses. Clostridiales and Clostridia have been observed to promote the production of SCFAs, thereby participating in host immune and anti-inflammatory responses by mediating the differentiation and generation of regulatory T cells (Treg cells)^35^. Ruminococcaceae belongs to Clostridia and constitutes a significant component of the gut microbiome present in the gastrointestinal tract of humans and animals. The primary functions of the bacterium include the production of SCFAs, binding and transporting mucins, and the degradation of carbohydrate components^36^. Members of the Ruminococcus genus, which have heretofore been linked to a healthy microbiome and less aggressive tumours, exhibited a significantly higher abundance in patients who derived clinical benefit from PhaseⅠ0 therapy^28^. Parasutterella, a constituent of the Proteobacteria phylum, constitutes a fundamental element of the human gut microbiome and has been linked to maladies such as fatty liver disease^37^. The Anaerostipes和Eubacterium__hallii_group are significantly enriched in HC, producing short-chain fatty acids with anti-inflammatory and anti-cancer properties, whose levels are reduced in patients with esophageal squamous cell carcinoma^38^. However, studies have also found that Eubacterium__hallii_group is associated with disease progression in patients with NSCLC^28^. It is noteworthy that the aforementioned gut microbiome exhibited high enrichment in healthy individuals, while lung cancer patients demonstrated significant depletion of several SCFAs-producing genera. This depletion may correspond to reduced SCFAs availability, thereby diminishing their protective effects on the host. This finding indicates that a decrease in butyrate and lactate production capacity may further contribute to intestinal barrier dysfunction in lung cancer. As this conclusion remains provisional, subsequent studies should incorporate serological or fecal metabolomic analyses to determine whether levels of SCFAs derived from the gut microbiome are significantly altered in LC.

The gut microbiome of LC patients has been shown to exhibit unique characteristics and composition. The present study identified significant enrichment of Bacilli, Ruminococcus_gnavus_group, and Prevotellaceae_NK3B31_groupin the intestines of LC patients. Despite the paucity of research conducted on bacilli, the genus Bacillus, which is part of the family under discussion, has been found to be enriched in cases of breast cancer. The findings of this study offer novel insights into the potential roles of these bacteria and the functional genes present in bacteria that generally promote metastasis, thereby highlighting their role in regulating disease progression^39^. Research indicates that the abundance of Ruminococcus_gnavus_group is reduced in diseases such as Parkinson disease^40^ and liver cirrhosis^41^. However, it has also been reported that *Ruminococcus gnavus* group inhibits TNF-α secretion, potentially suppressing lung cancer metastasis^42^. This finding suggests that the Ruminococcaceae family may exhibit differential levels and functions across various diseases. It has been hypothesised that this may serve as a specific microbial biomarker during certain stages of LC development, playing a significant pathophysiological role in both the initiation of lung cancer and the progression of LC metastasis. This has the potential to drive malignant progression of LC, though the precise regulatory mechanisms remain unclear. Prevotellaceae_NK3B31_group has been found to be enriched in HER2-positive breast cancer subtypes and is also associated with impaired glucose tolerance and obesity, potentially linking metabolic factors to cancer^22^. Intriguingly, the abundance of Prevotellaceae_NK3B31_group, a genus that produces butyrate, is diminished in patients with Lynch syndrome (LS)-associated CRC^43^. In the present study, the abundance of Prevotellaceae_NK3B31_group was found to be significantly elevated in patients diagnosed with LC. The findings demonstrate that the composition and dynamics of the gut microbiome in LC patients vary according to the pathological subtype and metastatic status of the cancer. This establishes the basis for a systematic, multifaceted assessment of the impact of the gut microbiome on LC.

Utilising 16S functional profiling, pronounced discrepancies in microbial functional abundance were identified between the two groups. The healthy control group exhibited a significantly broader spectrum of microbial functions, whereas LC patients showed elevated levels of expression in extracellular structures, as well as RNA processing and modification. During the course of the disease, a variety of metabolic capabilities of the gut microbiota were observed to be reduced (Table 2). In the gut microbiome of LC patients, the expression of functional proteins involved in defence mechanisms decreased by more than 9%, which may directly impacted the proliferation and colonization of gut bacteria. However, given that these findings are based on computational predictions, metabolomic and mechanistic validation is still required to confirm their relevance to the pathogenesis of lung cancer. The relationship between the gut microbiota and lung cancer patients is intricate, and currently, there is a paucity of definitive evidence; the human host and its gut microbiota interact and influence one another. In the context of disease, alterations in the composition and characteristics of the bacterial communities within the gut microbiota have been observed in the LC group. This may involve the suppression of beneficial effects and the enhancement of harmful functions, leading to alterations in the molecular structure and function of the gut microbiota, which could potentially influence the development of lung cancer.

## In conclusion

The present findings lend support to the hypothesis regarding the specific microbiome associated with LC; the gut microbiome of LC patients is altered, and the Prevotellaceae_NK3B31_group and Ruminococcus_gnavus_group may serve as potential diagnostic markers for LC. It was further demonstrated that there is a potential correlation between impaired normal gut microbiome function and the onset and progression of LC. It is hoped that the findings will provide a framework for the utilisation of the gut microbiome as biomarkers to assess LC progression or identify intervention targets to control disease development. Nevertheless, the question of whether gut microbiome alterations are a causative factor or a consequence of LC progression remains a subject of debate. The specific pathogenic mechanisms of LC, involving disease-associated molecular patterns and gut microbial metabolites, require further investigation. The present study is subject to several objective limitations. Firstly, the relatively limited sample size of this single-centre cross-sectional study may impede its capacity to discern more subtle or subtype-specific microbial distinctions. Secondly, variations in the genetic background, dietary habits, and environmental exposures of the study participants limit the statistical power and generalizability of the results. Thirdly, the 16S rRNA gene sequencing method is known to have limitations in determining microbial composition, which may lead to under detection and biased results.

## Supplementary Information

Below is the link to the electronic supplementary material.

Supplementary Material 1

## Data Availability

The datasets presented in this study are available in NCBI Sequence Read Archive (SRA) with the accession number PRJNA1406830.”And I have already released all these data, and they are now freely accessible.
