# Supplementary material for: Exploring the characteristics of gut microbiome changes in lung cancer patients and healthy controls
Source: Sci Rep. 2026 Apr 18;16:18051. doi: 10.1038/s41598-026-48560-w (PMC13253839; doi:10.1038/s41598-026-48560-w)
Supplement: Supplementary file 1 — Supplementary Material 1 [file 41598_2026_48560_MOESM1_ESM.docx]

**Table S1|Composition ratios of the top four bacterial groups in the gut microbiome of the LC group and the control group.**

| Taxonomy | LC(%) | Control(%) |
| --- | --- | --- |
| Bacteroidetes | 45.61 | 44.01 |
| Firmicutes | 33.19 | 42.21 |
| Proteobacteria | 15.83 | 9.87 |
| Actinobacteria | 2.91 | 1.87 |
